# Detection of oligoclonal IgG kappa and IgG lambda bands in cerebrospinal fluid and serum with Hevylite™ antibodies. comparison with the free light chain oligoclonal pattern

**DOI:** 10.1186/2045-8118-9-5

**Published:** 2012-02-23

**Authors:** David Zeman, Pavel Hradílek, Zdeněk Švagera, Eva Mojžíšková, Ivana Woznicová, Olga Zapletalová

**Affiliations:** 1 Institute of Clinical Biochemistry, University Hospital Ostrava, 17. Listopadu 1790, 708 52 Ostrava-Poruba, Czech Republic; 2 Clinic of Neurology, University Hospital Ostrava, 17. Listopadu 1790, 708 52 Ostrava-Poruba, Czech Republic

**Keywords:** Cerebrospinal fluid, Oligoclonal bands, IgG kappa, IgG lambda, Free light chains, Hevylite™

## Abstract

**Background:**

Oligoclonal IgG bands in cerebrospinal fluid that are absent in serum indicate intrathecal IgG synthesis and are a sensitive marker of CNS inflammatory diseases, in particular multiple sclerosis. It may be of interest to determine whether these bands are predominantly IgGκ or IgGλ.

**Methods:**

We have used Hevylite™ antibodies and developed a technique for detection of oligoclonal IgGκ and IgGλ bands by means of isoelectric focusing followed by immunoblotting. The same technique was used for oligoclonal free κ and free λ detection. Among several techniques tested, affinity immunoblotting appears to be the most sensitive; it can detect less than 1 ng of IgGκ or IgGλ paraprotein. We compared oligoclonal IgG profiles with those of oligoclonal IgGκ and IgGλ. There was good agreement concerning the presence or absence of intrathecal synthesis. We observed the ratios between oligoclonal IgGκ and IgGλ bands, and they did not always match the ratios between free κ and free λ bands. We were also able to detect antigen-specific CSF-restricted oligoclonal IgGκ and IgGλ bands in neuroborreliosis. It remains to be determined subsequently by a clinically-oriented prospective study, whether predominant IgGκ/IgGλ or free κ/free λ can be observed more frequently in particular diseases with oligoclonal IgG synthesis.

**Discussion:**

Very sensitive detection of oligoclonal IgGκ and IgGλ bands in cerebrospinal fluid with Hevylite antibodies is feasible; detection of antigen-specific IgGκ or IgGλ is possible as well. In particular situations, e.g. when difficulties arise in distinguishing between oligoclonal and monoclonal pattern, the test may be of considerable clinical value.

## Background

Oligoclonal IgG bands (OCBs) in cerebrospinal fluid (CSF) that are absent in serum indicate intrathecal IgG synthesis and are a sensitive marker of CNS inflammatory diseases, in particular multiple sclerosis (MS). Isoelectric focusing (IEF) and specific IgG detection by means of immunofixation (IF) or immunoblotting (IB) is the recommended procedure for OCBs detection [1-3]. It may be of interest to determine whether these bands are predominantly IgGκ or IgGλ. Previous reports used either quantitative tests [4-7] or immunofixation with kappa and lambda antisera [8-10] to demonstrate altered kappa/lambda or oligoclonal kappa/lambda ratios. However, these tests were not able to directly distinguish individual heavy-light chain pairs. Quantitative tests for total kappa and lambda light chains were neither specific nor sensitive enough for MS, and their use in CSF laboratories was almost completely abandoned. In 1989, Araga *et al*. described the quantitation of kappa/lambda ratios of each immunoglobulin (IgG, IgA, and IgM) in CSF and in sera by means of sandwich enzyme-linked immunosorbent assay [11]. Since the introduction of sheep Hevylite™ antibodies that target epitopes at junctions of the heavy chain and light chain constant regions, simple single-step analysis of heavy chain-light chain pairs has been available. The antibodies were developed for turbidimetric or nephelometric measurements in human sera. Heavy light chain serum immunoassays are used in patients with multiple myeloma and monoclonal gammopathy of undetermined significance (MGUS); they can provide quantitative information and be of prognostic significance in these patients [12,13]. In our opinion, these antibodies could be useful for CSF OCB detection and characterization. Hevylite antibodies were kindly provided by Dr. L. Assi and Dr. J. Faint (The Binding Site Ltd., Birmingham, United Kingdom) and we have developed a technique for the detection of oligoclonal IgGκ and IgGλ bands. Patterns observed in paired CSF and serum samples were compared with patterns of total IgG as well as with the patterns of free light chains (fLC).

## Methods

### Biotinylation of Hevylite and anti-human free light chain antibodies

Biotinylation of a portion of Hevylite antibodies and of antibodies against human free kappa and free lambda light chains (DAKO, Glostrup, Denmark, Cat. No. A0100 and A0101, respectively) was performed using EZ-Link^® ^NHS-PEO_4 _Biotinylation Kit (Pierce/Thermo Scientific, Rockford, USA, Cat. No. 21455). Hevylite antibodies were diluted to 1.0 g/l in phosphate-buffered saline (PBS) and proceeded further with buffer exchange into PBS and biotinylation. As a result, biotinylated antibody concentrations fell to 0.8 g/l approximately (due to the application of the stacker on gel filtration columns). Based on initial results with dilutions of biotinylated antibodies 1/200 and 1/1000, dilutions 1/500 for IgG kappa and 1/800 for IgG lambda were chosen for further experiments. Biotinylation of fLC antibodies required no prior buffer exchange since these antibodies were known to be supplied in PBS; these antibodies were diluted to 4 g/l in PBS for biotinylation, and biotinylated antibodies were used diluted 1/500 in our assay.

### Isoelectric focusing

IEF was performed in 0.5 mm thick agarose gel (1.1% agarose, 12% sorbitol, and either 6.4% Pharmalyte pH 3-10 or 6.2% Pharmalyte pH 3-10 plus 1.7% Pharmalyte pH 8-10.5), without prefocusing, using Multiphor II apparatus (GE Healthcare, Uppsala, Sweden). Interelectrode distance was 8.5 cm. 1700 V, 100 mA, and 10 W were used as limit values. Samples were applied 2 cm from the anode into the slots of Servalyt Precotes applicator strips 3.5*2 mm (Serva Electrophoresis GmbH, Heidelberg, Germany, Cat. No. 42899). IEF was run at 10°C for 1200 Vh (65-70 min).

### Affinity-mediated immunoblotting (AIB)

Nitrocellulose (NC) membranes (Amersham™ Hybond™-ECL, GE Healthcare, Cat. No. RPN303D, pore size 0.45 μm) were coated for 4-8 h with goat anti-human IgG Fc antibody (AbD Serotec, Oxford, UK, Cat. No. 5211-8004) diluted to 20 mg/l. Subsequently, the membranes were rinsed in tris-buffered saline pH 7.6 (TBS) and blocked with 3% bovine serum albumin (BSA, Serva Electrophoresis GmbH, cat. No. 11924) in TBS for 45-50 min, rinsed in TBS again and laid over the gel, followed by 1 sheat of filter paper moistened in TBS and 8 sheets of dry filter paper, a glass plate and a weight (approximately 0.5 kg). Affinity-mediated blotting was carried out for 50 min. Membranes were then washed in TBS and protein fixed with glutardialdehyde (0.25% in PBS) for 15 min at 4°C. After washing the membranes three times in TBS, the membrane was re-blocked with 0.3% BSA in TBS for 5-10 min, washed again in TBS, and biotinylated Hevylite antibody was applied (dilution in TBS with 0.3% BSA 1/500 for IgG kappa and 1/800 for IgG lambda) for 75 min. Membranes were washed once in TBS, twice in TBS-0.05% Tween20 (Serva Electrophoresis GmbH, Cat. No. 37470) (TBST), and once in TBS. Next, alkaline phosphatase streptavidin (Vector Laboratories Inc., Burlingame, USA, Cat. No. SA-5100) diluted 1/900, was applied for 50 min. After washing once in TBS, twice in TBST, and twice in TBS, colour reaction was performed using BCIP/NBT Kit (Vector Laboratories Inc., Cat. No. SK-5400) until suitable staining developed (15-25 min). Finally, the membranes were washed in TBS and deionized water, and dried between two sheets of filter paper.

For the analysis of total oligoclonal IgG, the procedure was the same up to the incubation with labeled antibody; in this case, we used alkaline phosphatase-conjugated anti-human IgG Fc antibody (AbD Serotec, Oxford, UK, Cat. No. 5211-8104) diluted 1/900 for 75 min and after washing procedure colour reaction was performed exactly as described above.

For the analysis of *Borrelia*-specific IgG, IgGκ, and IgGλ, membranes were coated with commercially available sonicated *Borrelia burgdorferi *organisms (AbD Serotec, Cat. No. 1440-0006) diluted in TBS to 20 mg/l. Further steps of the procedure were the same as described for total oligoclonal IgG, IgGκ, and IgGλ AIB analysis, except the incubation with biotinylated antibody (90 min) and colour development (about 30 min) was somewhat longer.

Oligoclonal fLC were analyzed by coating the membrane with anti-human kappa or lambda fLC (DAKO, Cat. No. A0100 and A0101, respectively), diluted to 20 mg/l in TBS. The following steps up to the incubation with the second antibody were exactly as described above. In-house biotinylated anti-human kappa or lambda fLC antibodies were used for detection (incubation time was 90 min for fLC OCBs detection), followed by the incubation with alkaline phosphatase streptavidin (see above). Colour development was longer for fLC than for IgG (about 30 min). For the estimation of assay sensitivity, commercially available purified human Bence Jones kappa and lambda free light chains (Bethyl Laboratories Inc., Montgomery, USA, Cat. No. P80-126 and P80-127, respectively) were used.

### Classical immunoblotting

#### a) Method with biotinylated Hevylite antibody and alkaline phosphatase streptavidin

Membranes were pre-wetted in deionized water (3-5 min) and then in TBS (30-45 min) and laid over gel, followed by 1 sheat of filter paper moistened in TBS and 8 sheets of dry filter paper, a glass plate and a weight. Blotting was carried out for 30 min. After washing in TBS, the membranes were blocked with 3% BSA for 60 min and washed 3 times in TBS. Next, biotinylated Hevylite antibody was applied (dilution 1/500 for IgG kappa and 1/800 for IgG lambda) for 75 min. Subsequent washing and detection were performed exactly as in the method above.

#### b) Double antibody method

Membranes were pre-wetted in deionized water (3-5 min) and then in TBS (30-45 minutes) and laid over gel, followed by 1 sheat of filter paper moistened in TBS and 8 sheets of dry filter paper, a glass plate and a weight. Blotting was carried out for 30 min. After the washing in TBS, the membranes were blocked with 3% BSA for 60 min, washed 3 times in TBS and Hevylite antibody was applied (diluted to 1.6 or 8 mg/l for IgG kappa and 1 or 5 mg/l for IgG lambda) for 75 min. Membranes were then washed once in TBS, twice in TBST, and once in TBS. Alkaline phosphatase-conjugated AffiniPure rabbit anti-sheep IgG, Fc Fragment Specific (Jackson ImmunoResearch Europe Ltd., Suffolk, UK, Cat. No. 313-055-046) was applied for another 75 min. Membranes were then washed once in TBS, twice in TBST, and twice in TBS. Colour development was performed as described above.

### Routine CSF analyses

For all samples, albumin, IgG, IgM and IgA were measured in CSF and serum by means of immunonephelometry on Immage analyzer (Beckman Coulter Inc., Brea, USA). Oligoclonal IgG analysis was performed using a commercial kit on Hydrasys instrument (Sebia, Évry Cedex, France, Cat. No. 4355) according to the manufacturer's instructions (except that the amount of the sample applied was 15 μl instead of 10 μl recommended in the instructions). Intrathecal immunoglobulin synthesis was calculated according to Reiber's formula [14]. Complete basic CSF diagnostics (cell count, differential cell count, total protein, lactate, and glucose) was also performed for all in-house patients.

### Samples

To assess the potential use of the method described, we analyzed 33 samples from 31 patients (MS, n = 5; clinically isolated syndrome, n = 3; neuroborreliosis, n = 5; AIDS with cerebral toxoplasmosis: n = 1; acute inflammatory demyelinating polyneuropathy, n = 2; other diagnoses, n = 15) for oligoclonal IgGκ and IgGλ; 1 patient was examined twice and 1 remaining sample was UK NEQAS quality control sample. Twenty-seven of these samples were also examined for oligoclonal fLC. *Borrelia*-specific IgG, IgGκ and IgGλ bands were searched for in 3 samples (1 case each of multiple sclerosis, neuroborreliosis, and neuroborreliosis with coincidental MGUS). For oligoclonal IgG, IgGκ and IgGλ analysis, samples were diluted to 5 mg/l IgG; for the analysis of *Borrelia*-specific OCBs, samples were diluted to 30 mg/l IgG and 6 μl of diluted sample was applied per lane. For oligoclonal fLC detection, CSF samples were used undiluted, serum samples were diluted 1/100, and 7.5 μl was applied per lane. All dilutions were performed in 0.8% saline. Only surplus of CSF and sera after a routine CSF analysis were used. Diagnoses were searched for in hospital records. The patient mentioned in the *Case Report *section gave written informed consent with the publication of his clinical and laboratory data. Analysis of a larger number of consecutive paired CSF and serum samples will follow in a prospective manner to avoid any possible bias. All samples will be analyzed for IgGκ, IgGλ, free κ and free λ OCBs and the results will be compared with clinical data as well as with the data from the routine CSF analyses.

The study was approved by the local Ethics Committee (Reference number 615/2011).

## Results of preliminary experiments

**Figure 1:**
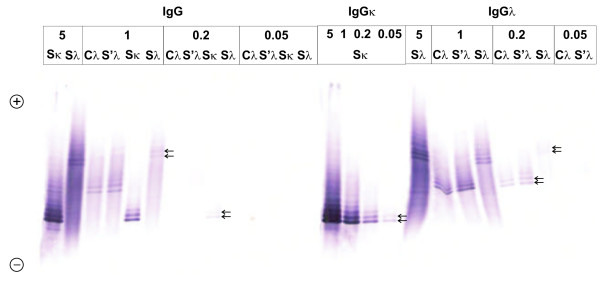
**Affinity immunoblots to estimate the detection limit of the IEF/AIB using several IgG paraproteins**. From left to right: total IgG, IgGκ, IgGλ. Based on serum protein electrophoresis and densitometry, serum IgGκ paraprotein (Sκ) represented 67.6% of total IgG, and serum IgGλ paraprotein (Sλ) represented 18.6% of total IgG. Using Hevylite antibodies, Sκ is clearly visible at an IgG concentration 0.05 mg/l, i.e. 0.3 ng of IgG or 0.2 ng of the paraprotein; Sλ is clearly visible at an IgG concentration 0.2 mg/l, i.e. 1.2 ng of IgG or 0.2 ng of the paraprotein (arrows). In total IgG OCB assay, the detection limit is several times higher but still very good (not higher than 1 ng of paraprotein). The percentage of paraprotein from total IgG was not known for paired CSF (C) and serum (S') sample (UK NEQAS Control 8/2011). The anode is at the top. A mixture of 6.2% Pharmalyte pH 3-10 and 1.7% Pharmalyte pH 8-10.5 was used for isoelectric focusing.

Affinity-mediated immunoblotting was evaluated as the most sensitive among the three techniques used. IgGκ and IgGλ paraproteins were detected in samples containing total IgG at a concentration of 0.2 mg/l, but even at the concentration of 0.05 mg/l faint bands were still observed. Biotin-streptavidin amplification step contributed to the sensitivity of the method because the test for "total" IgG failed to detect 0.05 mg/l concentration and only faint bands were seen at 0.2 mg/l (Figure 1). This, however, is of little importance for IgG. Much more important is to achieve high sensitivity detection for free κ and free λ light chains. Initially, we tried to use polyvinylidene difluoride (PVDF) membranes instead of nitrocellulose but this added little to the sensitivity of the method. Additionally, for unknown reasons we observed a high background on oligoclonal free λ light chain PVDF membranes, which was not observed on PVDF membranes tested for free κ, nor on nitrocellulose membranes tested for free κ or free λ. Therefore, we decided to use nitrocellulose membranes universally for all tests. We have tested commercially available purified human Bence Jones kappa and lambda free light chains and were able to detect 0.375 ng of free κ and 1.5 ng of free λ protein.

To assess reproducibility, the same samples were analyzed four times, twice in gel containing 6.2% Pharmalyte pH3-10 and 1.7% Pharmalyte pH 8-10.5, and twice in gel containing 6.4% Pharmalyte pH 3-10 only. These samples were: Sebia CSF control (Sebia, Cat. No. 4794) as a positive control (found to contain two groups of monoclonal-like bands, one each of IgGκ and IgGλ), intravenous immunoglobulin (Kiovig, Baxter S.A., Lessines, Belgium) as a negative control, one paired CSF (containing several faint IgG OCBs on IEF/IF) and serum (with no OCBs) sample, and two serum samples containing paraprotein of the IgGκ type and oligoclonal IgGκ, IgGλ, and free κ bands, respectively. OCBs pattern was slightly different depending on the pH gradient used. Regarding the presence or absence of bands, however, the results remained the same in all runs, except for the case of several (three to four) faint CSF-restricted IgGκ bands that were barely visible in two runs when only Pharmalyte pH 3-10 was used. On the other hand, the bands were clearly visible in both the runs using Pharmalyte 3-10 plus Pharmalyte pH 8-10.5. Several CSF-restricted IgGλ bands in this sample were clearly visible in all four runs. No cross-reactivity of the serum IgGκ paraprotein was seen on the membrane tested for IgGλ; several free κ bands in this sample had different migration positions than IgGκ bands.

**Figure 2:**
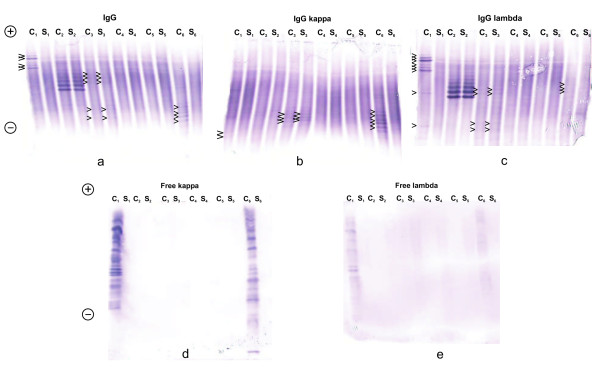
**Isoelectric focusing/affinity-mediated immunoblotting analysis of IgG (a), IgGκ (b), IgGλ (c), free κ (d), - and free λ (e) OCBs in 6 paired CSF (C) and serum (S), C_1 _-C_6_, S_1_- S_6_, cases 9, 33, 29, 30, 31, and 10 (Table 1)**. CSF 1 and CSF 6 were positive in all tests; however, the majority of IgG bands were of the lambda type in CSF 1, whereas of the kappa type in CSF 6 (arrowheads). Numerous free κ and free λ bands were present in CSF 1, whereas more free κ than free λ bands were present in CSF 6. Sample 2 is IgGλ paraprotein clearly detected using Hevylite IgGλ with no cross-reaction with IgGκ. Sample 3 represents "type 4", i.e. bands of equal intensity in CSF and serum that are both of IgGκ and IgGλ type (arrowheads). No intrathecal synthesis of IgG and no free light chain bands were detected. Finally, samples 4 and 5 represent normal (negative) results with no clear oligoclonal bands. Anode is at the top. A mixture of 6.2% Pharmalyte pH 3-10 and 1.7% Pharmalyte pH 8-10.5 was used for IgG, IgGκ, and IgGλ, whereas 6.4% Pharmalyte was only used for free κ and free λ.

One case of IgGλ paraprotein was observed that had initially been detected on Sebia Hydrasys by IEF followed by immunofixation. The sample was from NEQAS Quality Assurance for oligoclonal IgG and declared IgG concentrations were 4.0 mg/l in CSF and 1284 mg/l in serum. Interpretation on Sebia Hydrasys was possible, but more difficult than with AIB. Both AIB tests detected this paraprotein without any doubt, while the test for IgGλ gave clearer results than the test for "total" IgG (Figure 2).

No cross-reaction for free light chains was seen using intravenous immunoglobulin preparation (Flebogamma, Instituto Grifols, Barcelona, Spain) at a concentration of 250 mg/l.

**Figure 3:**
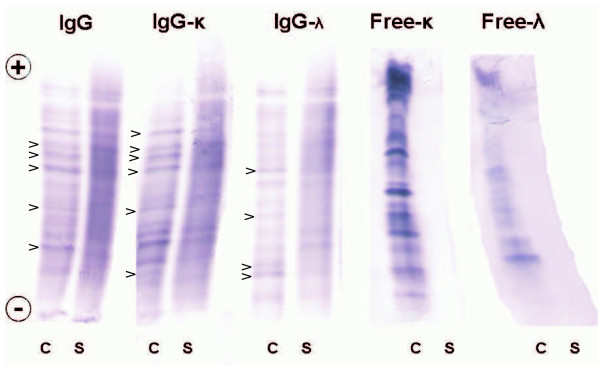
**Isoelectric focusing/affinity-mediated immunoblotting analysis of IgG, IgGκ, IgGλ, free κ, and freeλ OCBs in a patient with multiple sclerosis (Case 4 in Table 1) using paired CSF (C) and serum (S) samples**. Several CSF-restricted OCBs that were not seen in serum samples (arrowheads). Anode is at the top. Samples were analyzed on the same gel using a mixture of 6.2% Pharmalyte pH 3-10 and 1.7% Pharmalyte pH 8-10.5.

IF, immunofixation (Sebia Hydrasys); AIB, affinity immunoblotting; MS, multiple sclerosis; CIS, clinically isolated syndrome; MRI, magnetic resonance imaging; NB, neuroborreliosis; MGUS, monoclonal gammopathy of undetermined significance; AIDS, acquired immune deficiency syndrome; AIDP, acute inflammatory demyelinating neuropathy; CT, computed tomography; UK NEQAS, United Kingdom National External Quality Assurance Scheme

The analysis of paired CSF and serum samples revealed the presence of CSF-restricted IgGκ, IgGλ, free κ, and free λ bands in 12/33, 12/33, 16/27, and 11/27 samples, respectively (Table 1). An example of a positive result in all tests in an MS patient is given in Figure 3. The presence of OCBs was highly intercorrelated. Among 13 cases positive for IgGκ or IgGλ OCBs, both types were seen in 11 cases, the remaining 2 cases having IgGκ or IgGλ only. One patient was negative for IgG OCBs (IEF/IF), but positive when tested with both Hevylite antibodies (Case No. 14). Numerous identical bands were seen in CSF and serum; however, Hevylite antibodies revealed some bands being much stronger in CSF than in serum. In one additional patient (Case No. 12), no OCBs were revealed by IEF/IF, but 1 band was observed when tested for total IgG and for IgGκ by means of IEF/AIB. Based on recommendations of the German society for CSF diagnostics and clinical neurochemistry [15], this was considered to be a negative result. All cases showing CSF-restricted OCBs with Hevylite antibodies displayed also free κ OCBs; these were, however, observed in 6 cases with negative IgG OCBs (including 1 borderline result of 1 IgGκ band mentioned above). Among 16 samples positive for fLC OCBs, both fLC OCBs were detected in 11 cases and only free κ OCBs in the remaining 5 cases.

**Table 1:** CSF-restricted oligoclonal IgG, IgGκ, IgGλ, free κ and free λ bands

**No**.	Diagnosis	Cells/μl	Albumin quotient (*10^3^)	Intrathecal Ig fraction (%)	IgG(IF) (type)*	IgG(AIB)	IgGκ	IgGλ	fκ	fλ	IgGκ/IgGλ	fκ/fλ
1	MS	5	2.5	IgG 36%; IgM 30%	+ (type 3)	n.d.	+	+	+	+	=	=

2	MS	62	9.6	IgG 65%	+ (type 2)	n.d.	+	0	n.d.	n.d.	IgGκ only	n.d.

3	MS	5	2.9	IgG 30%, IgM 43%	+ (type 2)	n.d.	+	+	+	+	=	fκ > fλ

4	MS	5	4.5	IgG 65% IgM 77%	+ (type 3)	+	+	+	+	+	=	=

5	MS	1	3.1	0	+ (type 3)	n.d.	+	+	n.d.	n.d.	=	n.d.

6	CIS	6	3.4	0	+ (type 2)	n.d.	0	+	+	+	IgGλ only	fκ > fλ

7	CIS	3	2.7	0	+ (type 3)	n.d.	+	+	+	+	=	fκ > fλ

8	CIS	1	3.3	0	+ (type 2)	+	+	+	+	+	IgGλ > IgGκ	=

9	NB	118	37.4	IgM 70%	+ (type 2)	+	+	+	+	+	IgGλ > IgGκ	=

10	NB	469	30.0	IgM 40%	+ (type 2)	+	+	+	+	+	IgGκ > IgGλ	fκ > fλ

11	NB + MGUS	145	30.0	IgG 30% IgM 54%	+** (type 3 + type 5)	+**	+**	+	+	+	IgGλ > IgGκ	fκ > fλ

12	NB	35	12.8	0	0 (type 1)	0***	0***	0	+	0	1 IgGκ band only	fκ only

13	NB	100	32.8	IgM 42%	+ (type 3)	+	+	+	n.d.	n.d.	=	n.d.

14	AIDS, cerebral toxoplasmosis	7	10.5	IgM 53%	0 (type 4)	n.d.	+	+	+	+	=	fκ > fλ

15	AIDP	3	11.2	0	0 (type 4)	0	0	0	+	0	negative	fκ only

16	AIDP -1st puncture	1	51.5	0	0 (type 4)	0	0	0	+	+	negative	fκ > fλ

17	AIDP - 2nd puncture	1	39.4	0	0(type 4)	0	0	0	+	0	negative	fκ only

18	Oculomotor nerve paresis	3	6.7	0	0 (type 1)	n.d.	0	0	n.d.	n.d.	negative	n.d.

19	Neurodegenerative (parkinsonism)	2	6.2	0	0 (type 1)	0	0	0	+	0	negative	2 weak fκ bands

20	Tension headache	5	6.4	0	0 (type 1)	0	0	0	0	0	negative	negative

21	Dorsalgias, myalgias	0	5.3	0	0 (type 1)	0	0	0	0	0	negative	negative

22	Memory disturbance	0	3.0	0	0 (type 1)	0	0	0	0	0	negative	negative

23	Acute central hemiparesis with complete regression (post-paroxysmal?; normal brain MRI)	0	4.0	0	0 (type 4)	0	0	0	0	0	negative	negative

24	Posterior columns syndrome, unknown etiology	3	21.0	IgM 12%	0 (type 1)	0	0	0	+	0	negative	fκ only

25	Lacunar stroke	1	3.9	0	0 (type 1)	n.d.	0	0	n.d.	n.d.	negative	n.d.

26	Gait disturbance, unknown aetiology	0	4.9	0	0 (type 1)	n.d.	0	0	n.d.	n.d.	negative	n.d.

27	Orthostatic hypotension (normal brain MRI)	1	7.5	0	0 (type 1)	n.d.	0	0	0	0	negative	negative

28	Headache (brain CT negative)	0	3.2	0	0 (type 4)	n.d.	0	0	0	0	negative	negative

29	Vertigo	1	11.3	0	0 (type 4)	0	0	0	0	0	negative	negative

30	Peripheral facial palsy	1	4.0	0	0 (type 1)	0	0	0	0	0	negative	negative

31	Lyme arthritis	2	4.2	0	0 (type 4)	0	0	0	0	0	negative	negative

32	Polyneuropathy, lumbar disc prolapse	1	7.4	0	0 (type 4)	0	0	0	0	0	negative	negative

33	Paraprotein (UK NEQAS sample 8/2011)	n.d.	n.d.	n.d.	0**** (type 5)	0****	0	0****	0	0	negative	negative

* IEF pattern type according to the international consensus classification [1,15]

** paraprotein IgGκ detected

*** the presence of only 1 CSF-restricted band was considered to be a negative finding

**** paraprotein IgGλ detected

Intrathecal Ig (IgG, IgM, IgA) fraction has been assessed according to Reiber's formula [14].

n.d., not determined; +, positive (≥ 2 CSF-restricted bands); 0, negative; = , no clear preponderance of a particular IgG-bound or free light chain type

*Borrelia*-specific IgG, IgGκ and IgGλ bands were found in one patient with neuroborreliosis, while the other patient tested had only 2 clear-cut IgGλ *Borrelia*-specific bands. No clear-cut bands were seen in one patient with multiple sclerosis.

## Case report

**Figure 4:**
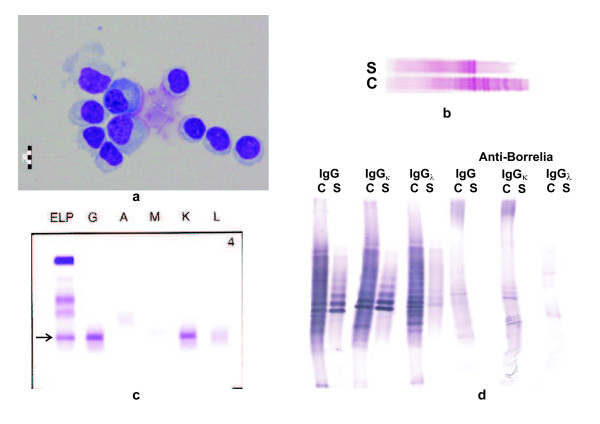
**Case report (case 11 in Table 1): CSF (C)and serum (S) findings**. 4a. Lymphocytic pleocytosis and 2 plasma cells in the CSF cytospin preparation. 4b. Isoelectric focusing/immunofixation using a commercial kit on Hydrasys instrument (Sebia) revealed CSF-restricted oligoclonal IgG bands as well as identical paraprotein bands in CSF and serum. Anode is on the left. 4c. Serum immunofixation electrophoresis (Sebia) revealed the presence of IgGκ monoclonal band (arrow). Anode is at the top. ELP, electrophoresis (general protein stain); G, A, M, K, L: immunofixation for γ, α, μ, κ and λ chain, respectively. 4 d. Isoelectric focusing/affinity-mediated immunoblotting analysis of case 11: Paraprotein bands did not react with *Borrelia *antigen but partly obscured CSF-restricted oligoclonal IgG pattern. Paired CSF and serum samples tested for (from left to right) "total" IgG, IgGκ, IgGλ; Anti-Borrelia IgG, IgGκ, IgGλ. Anode is at the top. Samples were analyzed on the same gel using a mixture of 6.2% Pharmalyte pH 3-10 and 1.7% Pharmalyte pH 8-10.5.

A 69-year-old male was admitted to our hospital with the diagnosis of acute inflammatory demyelinating polyneuropathy (AIDP), based on clinical and EMG findings. At the neurological exam the patient presented himself with progressive flaccid quadriparesis and bilateral facial peripheral palsy. However, CSF analysis revealed lymphocytic pleocytosis (145 cells/μl with 85% lymphocytes, 2% plasma cells, 11% monocytes, and 2% neutrophils; Figure 4a), elevated total protein (1911 mg/l) and lactate (3.36 mmol/l), severe blood-CSF barrier dysfunction (Q-Albumin 30.0*10^-3^, i.e. 3.5 times the upper age-related reference limit), and intrathecal synthesis of IgG and IgM (but not IgA) according to Reiber's formula (IgG, 30% and IgM, 54%), a pattern highly suggestive of neuroborreliosis that was confirmed serologically (IgG antibody index 33.5). Routine analysis of IgG OCBs on Sebia Hydrasys instrument revealed numerous CSF-restricted OCBs and several faint OCBs identical in CSF and serum, but also intense paraprotein bands identical in CSF and serum (Figure 4b). The quantity of paraprotein was 6.6 g/l, as determined densitometrically after serum protein electrophoresis. Additionally, a mild decrease in polyclonal gamma globulins was observed. Immunofixation revealed IgG kappa monoclonal band (Figure 4c).

Unusual combination of neuroborreliosis and monoclonal gammopathy made us decide to analyze the patient's immunoglobulin profile in CSF and serum in more detail. Biotinylated Heavylite antibodies were used for the analysis of IgGκ and IgGλ oligoclonal bands, as well as *Borrelia*-specific OCBs. The analysis of total oligoclonal IgGκ and IgGλ showed IgGκ paraprotein both in CSF and in serum and CSF-restricted oligoclonal bands were predominantly of the IgGλ type. However, borrelia-specific oligoclonal bands were both of IgGκ and IgGλ type. (Figure 4d). We confirmed the presence of IgGκ paraprotein which did not react with *Borrelia *antigen, but probably partly obscured intrathecally synthesized oligoclonal bands. Both free κ and free λ light chain OCBs were detected in CSF but not in serum; no attempt was made to analyze possible free light chain OCBs reactivity with *Borrelia *antigen. Serum free light chain analysis revealed normal concentrations and a normal free κ/free λ ratio. Bone marrow aspirate revealed no malignant cells. After the treatment with antibiotics and physiotherapy the patient's clinical status improved: the patient is self-sufficient and able to walk with bilateral assistance.

## Discussion

Our modification of the IEF/AIB method, based on the AIB method originally described by Knisley and Rodkey [16] and adapted for CSF analysis by Kaiser [17] and Sindic and Laterre [18], uses a glutardialdehyde fixation step to increase sensitivity [19]. The method was further modified by using alkaline-phosphatase instead of peroxidase-based detection. When tested for total oligoclonal IgG analysis, our method exhibited better analytical sensitivity than the commercial immunofixation method (Sebia). However, this fact does not seem to add anything to the clinical sensitivity, as no significant difference was observed in the proportion of positive and negative samples between these two methods [20]. Nevertheless, lowering the detection limit is of crucial importance for oligoclonal fLC analysis, since these are present in much lower concentrations in the CSF.

Several authors have reported altered CSF kappa/lambda ratios [4-7,11,21] as well as predominance of kappa (using free and bound anti-light chain antisera) OCBs [8,10,22] in a proportion of patients with MS. Such conclusions could not be reached in our methodological study due to a low number of patients tested. However, we have observed that once oligoclonal IgG bands are present, they are usually of both IgGκ as well as IgGλ type and are always accompanied by free κ and often also by free λ OCBs. Free κ OCBs could rarely be seen even in the absence of IgG OCBs. The fact that free λ OCBs were less frequently observed could, however, be explained in part by apparently lower sensitivity of our free λ (compared to free κ) OCBs test. Differences in antibody avidity could also, at least partly, explain discrepancies among various reports.

Oligoclonal fLC bands of the kappa type are seen in MS at approximately the same frequency as oligoclonal IgG; oligoclonal free lambda light chains are less frequently observed [18,23]. Predominantly free κ and free λ light chain intrathecal synthesis has been reported in MS patients and in patients with neuroinfections, respectively [24]. Although several attempts at fLC quantitation in CSF have been reported and there is almost unique agreement that this test may be useful in the context of the diagnosis of inflammatory CNS diseases, MS in particular [24-31], absolute values measured are highly discrepant. This is the reason we still prefer the qualitative IEF method. The observation that preferential IgGκ or IgGλ synthesis does not always correspond to preferential free κ or free λ synthesis, respectively, is quite surprising.

To our pleasant surprise, Hevylite antibodies can detect antigen-specific oligoclonal IgG bands. Hence, it can be concluded that the recognition of epitopes at the junction of the heavy chain-light chain constant region is not affected when the IgG molecules to be detected bind antigen via their antigen-binding site. Except for the reproducibility experiment mentioned above, we have always used gels with alkaline extension of the pH gradient for IgGκ and IgGλ OCBs analysis, whereas free κ and free λ OCBs were analyzed deliberately with or without alkaline extension. Although no differences were noted, we will prefer to analyze free κ and free λ OCBs separately from IgGκ and IgGλ OCBs and without alkaline extension. Alkaline extension is not necessary for free κ and free λ. From the practical point of view, it is much more convenient to handle two membranes (tested for IgGκ and IgGλ in one gel, and for free κ and free λ in another) than to perform all analyses simultaneously.

The limits of our study include: 1. no attempt made to quantitate the relative amount of IgGκ/IgGλ and free κ/free λ was made; such an attempt was reported for free κ and free λ monomers and dimers [32], but it requires an appropriate software and remains inherently semi-quantitative at best, although it is probably better than simple visual inspection; 2. a low number of samples tested during preliminary experiments which were concentrated on methodology, with an attempt to show feasibility of the method to detect IgGκ and IgGλ OCBs using Hevylite antibodies and to look for a possibility to compare IgGκ/IgGλ with free κ/free λ profiles. 3. a need for more extensive reproducibility testing with the use of more paired CSF and serum samples analyzed at least twice in different runs.

Nevertheless, the test could be useful at least in some unusual circumstances, as demonstrated in our case report. Although monoclonal pattern (type 5) is easily discerned in typical cases, there are rare cases when difficulties may arise to distinguish it from type 4 (serum-derived oligoclonal bands). IgG light chain type and possibly the presence or absence of reactivity with presumed responsible microbial agent may provide a clue in such circumstances. In our case, polyradiculoneuritic syndrom was clearly attributable to neuroborreliosis, whereas monoclonal gammopathy of undetermined significance (MGUS) was an accidental finding. However, polyradiculoneuritis associated with multiple myeloma has been described [33] and could potentially be a differential diagnostic problem.

Another possible application of the method is testing for residual paraproteins in serum and/or urine of myeloma patients in complete remission (negative immunofixation electrophoresis), although we do not dare to extend this hypothesis to other Ig classes since the interpretation of IEF patterns is difficult for other monoclonal immunoglobulins than IgG.

Conversely, it would be very interesting to perform quantitative IgGκ and IgGλ tests with Hevylite antibodies in paired CSF and serum samples if a modification of this test for CSF becomes available.

Finally, it might be of interest to test for the reactivity of CSF-restricted oligoclonal fLC bands with microbial antigens. In our experience, CSF-restricted oligoclonal fLC are common in neuroborreliosis and there remains a possibility that oligoclonal fLC (dimers) may have antigen-binding capability [34,35], although this has been disputed by others [36]. In our case, this opportunity was missed.

In conclusion, we have adapted an affinity-mediated immunoblotting technique after IEF for the detection of IgGκ and IgGλ oligoclonal bands in cerebrospinal fluid and serum by means of Hevylite antibodies using biotin-streptavidin amplification step. We compared the results with those of oligoclonal free light chains tested by a similar method. The method enables the detection of IgGκ and IgGλ oligoclonal bands in the same way as for oligoclonal IgG, without the need of subsequent immunofixation and, presumably, without cross-reactivity with other immunoglobulins or free light chains. The method was able to detect less than 1 ng IgGκ, IgGλ or free κ light chain monoclonal protein and approximately 1.5 ng of free λ light chain monoclonal protein corresponding to the concentration of 0.2 mg/l for free λ light chains and even less for IgGκ, IgGλ and free κ light chains.

The proportion between IgGκ and IgGλ bands in individual patients is variable and does not always correspond with the proportion between free κ and free λ bands. It remains to be determined whether this test adds anything clinically useful to the routine oligoclonal IgG bands detection or whether predominant IgGκ/IgGλ or free κ/free λ pattern can be observed more frequently in certain diseases with oligoclonal IgG synthesis. The comparison (albeit only qualitative) of the intensity of oligoclonal IgGκ versus IgGλ as well as free κ versus free λ may be of theoretical interest. At least in some circumstances our method seems to be useful clinically, e.g. in rare cases where difficulties arise in the differentiation between type 4 (systemic oligoclonal pattern) and type 5 (monoclonal pattern).

## Abbreviations

AIB: affinity-mediated immunoblotting; AIDP: acute inflammatory demyelinating polyneuropathy; BCIP/NBT: 5-bromo-4-chloro-3-indolyl phosphate/nitroblue tetrazolium; BSA: bovine serum albumin; CIS: clinically isolated syndrome; CSF: cerebrospinal fluid; CNS: central nervous system; fLC: free light chains; IEF: isoelectric focusing; IB: immunoblotting; IF: immunofixation; IgA: immunoglobulin A; IgM: immunoglobulin M: IgG: immunoglobulin G; MGUS: monoclonal gammopathy of undetermined significance; MRI: magnetic resonance imaging; MS: multiple sclerosis; NB: neuroborreliosis; NC: nitrocellulose; n.d.: not determined; OCBs: oligoclonal bands; PBS: phosphate-buffered saline; PVDF: polyvinylidene difluoride; TBS: tris-buffered saline; TBST: TBS-0.05% Tween 20; UK NEQAS: United Kingdom National External Quality Assurance Scheme.

## Competing interests

The authors declare that they have no competing interests.

## Authors' contributions

DZ, PH and ZŠ designed the study. DZ performed and evaluated all immunoblotting experiments and wrote the first draft of the manuscript. PH, IW and OZ critically reevaluated clinical diagnoses in patients presented in Table 1 and discussed the possible clinical relevance of the tests described in the manuscript. EM was the treating physician of the patient mentioned in the case report and provided detailed clinical data. ZŠ critically reevaluated laboratory results and edited all figures. All authors reviewed and edited the manuscript and approved the final version of the manuscript.
